# Estradiol Increases Mucus Synthesis in Bronchial Epithelial Cells

**DOI:** 10.1371/journal.pone.0100633

**Published:** 2014-06-25

**Authors:** Anthony Tam, Samuel Wadsworth, Delbert Dorscheid, Shu-Fan Paul Man, Don D. Sin

**Affiliations:** The UBC James Hogg Research Centre, Providence Heart + Lung Centre & Department of Medicine, University of British Columbia (UBC), Vancouver, British Columbia, Canada; University of California, Merced, United States of America

## Abstract

Airway epithelial mucus hypersecretion and mucus plugging are prominent pathologic features of chronic inflammatory conditions of the airway (e.g. asthma and cystic fibrosis) and in most of these conditions, women have worse prognosis compared with male patients. We thus investigated the effects of estradiol on mucus expression in primary normal human bronchial epithelial cells from female donors grown at an air liquid interface (ALI). Treatment with estradiol in physiological ranges for 2 weeks caused a concentration-dependent increase in the number of PAS-positive cells (confirmed to be goblet cells by MUC5AC immunostaining) in ALI cultures, and this action was attenuated by estrogen receptor beta (ER-β) antagonist. Protein microarray data showed that nuclear factor of activated T-cell (NFAT) in the nuclear fraction of NHBE cells was increased with estradiol treatment. Estradiol increased NFATc1 mRNA and protein in ALI cultures. In a human airway epithelial (1HAE_0_) cell line, NFATc1 was required for the regulation of MUC5AC mRNA and protein. Estradiol also induced post-translational modification of mucins by increasing total fucose residues and fucosyltransferase (FUT-4, -5, -6) mRNA expression. Together, these data indicate a novel mechanism by which estradiol increases mucus synthesis in the human bronchial epithelium.

## Introduction

In most chronic inflammatory diseases of the airway (e.g, asthma, chronic obstructive pulmonary disease, and cystic fibrosis), female patients generally have more symptoms, worse quality of life and poorer prognosis than male patients [1], [2]. One pathological link that is common to these conditions is mucus hypersecretion with plugging of airways [3]. Mucins are large, oligomeric, O-linked glycoprotein with high molecular weight (2–40 MDa) and size (0.5–10 µm) [4]. As the predominant mucins in the human airway, MUC5AC is produced mostly by goblet cells, while MUC5B is produced mainly in submucosal glands [5], [6]. MUC5AC is thought to be an acute phase product that responds rapidly to direct contact with environmental insults, whereas MUC5B may be involved in the chronic inflammatory response to infections [7]. Mucin expression has been shown to be regulated by a variety of inflammatory mediators including lipopolysaccharide (LPS) [8], tumor necrosis factor (TNF)-α [9], interleukin (IL)-1 [9], IL-17 [10], IL-13 [11] β neutrophil elastase [12], and growth factors such as epidermal growth factor (EGF) [13], and environmental insults including cigarette smoke [14] and bacteria [15]. Even in genetically determined diseases such as cystic fibrosis, in which abnormal mucus secretion is a prominent feature of disease, lung function is the lowest at ovulation when estrogen levels are elevated [2], raising the possibility that estrogens may play an active role in this condition. Estrogen receptors alpha and beta exist in normal human bronchial epithelial (NHBE) cells with ER-β in predominance [16]. However, little is known whether estradiol plays any role in the regulation of mucus in the airways.

MUC5AC mRNA expression has been shown to be regulated by a variety of transcription factors including Forkhead box protein A2 (FOXA2), (NF
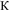
B), activating protein 1 (AP1), specificity protein (SP1) and cyclic AMP response element binding (CREB) protein [17]. In this study, we used an *in vitro* model of the human airway epithelium and showed that nuclear factor of activated T-cell c1 (NFATc1) is a novel regulator of estradiol-enhanced mucus synthesis.

## Materials

17-β estradiol was purchased from Sigma (St. Louis, MO). MPP [1,3-Bis(4-hydroxyphenyl)-4-methyl-5-[4-(2-piperidinylethoxy)phenol]-1H-pyrazole dihydrochloride] (ER-α antagonist), and PHTPP [4-[2-Phenyl-5,7-*bis*(trifluoromethyl)pyrazolo[1,5-*a*]pyrimidin-3-yl]phenol] (ER-β antagonist) were obtained from Tocris (Ellisville, MO). Antibodies against ER-α (ab2746), ER-β (ab3577), and MUC5AC (ab24071) were obtained from Abcam (Cambridge, MA). Anti-IgG (sc-2343), HRP-conjugated anti-β-actin (sc-47778), superoxide dismutase –SOD (sc-11407), histone H3 (sc-8655) and NFATc1 antibody (sc-7294) were obtained from Santa Cruz Biotechnology. Heat shock protein -HSP90 (610418) antibody was obtained from BD Biosciences (Mississauga, ON). Goat HRP-conjugated anti-mouse IgG was from BD Pharmingen (Franklin Lakes, NJ) and goat HRP-conjugated anti-rabbit IgG from Millipore (Billerica, MA).

## Cell cultures

The current study was approved by the University of British Columbia/Providence Health Care Research Ethics Board (Number H11-02151 and A13-0207). Written informed consent was obtained from subjects where appropriate. NHBE cells were purchased from Lonza (Walkersville, MD) and the International Institute for the Advancement of Medicine (IIAM, Jessup, PA) (N = 4 females, age: 16-45 yr). Initially, cells were seeded at passage 0 into T25 flasks in Bronchial Epithelial Growth Media (BEGM, Lonza) and sub-cultured when 90% confluent. Cells were seeded in an air liquid interface at passage 4 on a 0.4 µm semi-permeable membrane insert (BD Biosciences) in PneumaCult-ALI medium (StemCell Technologies, Vancouver, Canada). Apical media was removed when cells reached 100% confluence while media in the basal compartment was replaced 3 times per week. Cells at 21 days post ALI were fully differentiated, followed by treatment of physiological concentrations of estradiol (E2) (10^−9^ M to 10^−7^ M) for 2 weeks. Mucus overlying the epithelium in each well was washed with 500 µL warmed PBS and collected every 7 days. ALI cultures were pre-treated with hormone receptor antagonists 24 h prior to the beginning of experiment. 10^−6^ M MPP and 10^−6^ M PHTPP were added to the basal media and replaced 3 times per week. 0.01% final concentration of ethanol was used as a vehicle control. Human airway epithelial cell line (1HAE_0_) was obtained from Dr Dieter Gruenert University of California, San Francisco [18] and cultured in DMEM (Gibco BRL; Invitrogen, Carlsbad, CA) with 10% fetal bovine serum (FBS). Estradiol concentration in media containing 10% FBS was less then 0.2 pg/ml and was negligible compared to the estradiol concentrations added in culture.

## Histological staining

5 µm sections of paraffin-embedded human lung tissue from N = 4 pre-menopausal female subjects (age: 16–45 yr) with normal lung function and ALI cultures were stained for ER-α, ER-β, MUC5AC and Ki67 using the high sensitivity universal detection system-MACH kit (Biocare Medical, Concord, CA) (red). Sections were stained with Periodic Acid Schiff (PAS) for polysaccharides, nuclei were counterstained with Mayer's hematoxylin, dried overnight at room temperature and coverslipped in Cytoseal mounting solution. We used a point counting method to quantify the percentage of cells stained positively for the protein of interest by dividing the total number of epithelial cells counterstained with hematoxylin in three random fields at 200X final magnification from 4 different female subjects. PAS-positive cell count was based on counting nucleus to the nearest apical surface immediately beneath the PAS stain; whereas ciliated cells were determined by counting nucleus nearest to the apical surface in cells with cilia but without PAS staining. Both of these measurements were normalized to the length of the epithelium in millimeters. To ensure that we accurately quantify the amount of PAS staining, we also measured the area of the PAS stain and expressed this value as a percentage of the total epithelial cross sectional area using color segmentation (ImagePro Plus 4.0, Media Cybernetics, L.P). These experiments were performed in 4 female donors using 3 biological replicates. The data are shown as mean ± SEM.

## Transcription factor activation protein array

A transcription factor (TF) activation profiling plate array from Signosis (FA-1001; Sunnyvale, CA) was used to screen for activated TFs in nuclear protein extract from female primary NHBE cells (N = 2) stimulated with 10^−7^ M estradiol for 24 h. A mixture of biotin-labelled probes based on the consensus sequences of TF DNA-binding sites was incubated with nuclear protein extract for 30 min at room temperature and allowed to form TF/probe complexes. The TF/probe complexes were separated from free probes through a simple spin column purification step. The bound probes eluded from the column were denatured at 98°C for 5 min and allowed to hybridize with the biotin-conjugated consensus sequence initially bound on the bottom of each well in a 96-well plate. The captured DNA probe was detected with streptavidin-HRP. Luminescence was reported as relative light units on a microplate luminometer. Data were generated from 2 female donors.

## Real time PCR

RNA from ALI cultures and 1HAE_0_ cells was isolated using an RNeasy mini extraction kit from (Qiagen, Germantown, USA). High RNA quality in all samples was confirmed (RNA Integrity Number greater than 8) using an Agilent BioAnalyzer at The Centre for Applied Genomics (TCAG) in Toronto (data not shown). cDNA was synthesized with random hexamers using SuperScript III reverse transcriptase (Invitrogen, Life Technologies). TaqMan PCR probe-primers sets for FUT-1 (Hs00382532_m1), FUT-2 (Hs00704693_m1), FUT-3 (Hs01868572_m1), FUT-4 (Hs01106466_s1), FUT-5 (Hs00704908_s1), FUT-6 (Hs00173404_m1), FUT-7 (Hs00237083_m1), FUT-8 (Hs00189535_m1), FUT-9 (Hs00276003_m1) (Applied Biosystem-ABI, Carlsbad, CA), and NFATc1, NFATc2, NFATc3, NFATc4, MUC5AC and HPRT1 (sequences obtained from primerbank http://pga.mgh.harvard.edu/primerbank/and synthesized by Invitrogen) were used to create and quantify PCR products using an ABI 7900HT real-time qPCR machine using the Δ^CT^ method of relative quantification with HPRT1 as the housekeeping gene. mRNA expression were plotted as mean ± SEM. Data were generated from 4 different female donors with 3 biological replicates.

## Western blot analysis

For western blot analysis, ALI cultures were mechanically detached in ice-cold phosphate-buffered saline (PBS) using rubber cell scrapers, and pelleted by centrifugation at 17,000x gravity for 8 minutes. Cells were lysed in cytoplasmic extraction buffer (Thermo scientific, Ontario, CAN) for 30 minutes at 4°C on a rotating apparatus and the supernatant was collected and stored at −80°C. Nuclear protein was subsequently extracted in nuclear extraction buffer from the pellet after centrifugation according to the manufacturer. 15 µg of cellular protein lysates from ALI cultures were resolved by 10% SDS-PAGE and transferred to a nitrocellulose membrane (Millipore, Bedford, MA). Membranes were incubated with primary antibodies against ER-α, ER-β, NFATc1, MUC5AC, or (LTA-lotus tetragonolobus asparagus pea: H1601-1, AAA-anguilla anguilla lectin from fresh water eel: H4901-1, and UEA-1-ulex europaeus lectin from gorse: H2201-1) lectins (EY Laboratories Inc., San Mateo, CA) followed by incubation of secondary HRP-conjugated anti-rabbit, anti-mouse and beta-actin antibodies for 1 h at room temperature, and visualized using an enhanced chemiluminescence substrate (Thermo Scientific, Ontario, CAN) and a Chemigenius imaging system (Syngene, Cambridge, UK). Data were generated from 4 female donors.

## Small interfering RNA treatment

The role of NFATc1 in mediating the effect of estrogen in the transcription of MUC5AC was examined using small interfering (si)RNA siNFATc1 to silence NFATc1 mRNA. siNFATc1 (sc-156125) was purchased from Santa Cruz Biotechnology. The siRNA Universal Negative Control (SIC001) was used. 100 nM of siNFATc1, and siRNA negative control were used for transfection using Nanoparticle siRNA Transfection System (N2913) from Sigma according to the manufacturer's procedures. 1HAE_0_ cells were transfected for 48 h prior to estradiol stimulation at 10^−7^ M for 24 h, followed by harvesting for RNA and protein. Data were generated from 3 independent experiments with 3 biological replicates.

## Cytotoxicity

An *In Situ* Apoptosis Detection Kit was used to detect apoptotic cells in paraffin-embedded ALI culture sections (Trevigen; Gaithersburg, MD). We used a TACS•XL-DAB *In Situ* Apoptosis Detection Kit to detect nuclear DNA fragmentation as an indicator of cell apoptosis. This is performed *in situ* by incorporating labeled nucleotides (BrdU) onto the free 3′-OH ends of DNA fragments using a terminal deoxynucleotidyl transferase enzyme (TdT). Biotinylated anti-BrdU antibody and streptavidin-HRP were used for visualization of DNA fragmentation. Apoptotic cells were stained in brown and nuclei were counterstained with methyl green. Data were generated from 4 female donors. 100% cigarette smoke extract (CSE) was generated by bubbling smoke from 3 research grade (3R4F) cigarettes (University of Kentucky; Lexington, KY) via a 50 ml syringe into 5 ml of PBS solution. 1HAE_0_ cells were exposed to a final concentration of 10% CSE for 24 h and cell supernatant was used as positive control for lactate dehydrogenase (LDH) assay. LDH cytotoxicity detection kit (Cat. No. 11644793001, Roche Applied Science) was used to determine % cell death in cell supernatant of 1HAE_0_ cells that were exposed to 100 nM siNFATc1 for 48 h. Please refer to LDH assay kit methods for more detail. A final concentration of 0.2% Trypan blue solution (15250-061, Life Technologies) was added to cells and incubated for 2 minutes and imaged for indication of cell death.

## Statistical analysis

For statistical analysis of histochemical staining, immunohistochemistry and western blotting, non-parametric t-test was used to compare between two groups. One-way ANOVA with Bonferroni's test was used to account for multiple comparisons where appropriate. Statistical significance for all analyses was considered at *P<*0.05 using GraphPad Prism version 5 (GraphPad Software Inc, San Diego, CA, USA). All data values are expressed as mean ± standard error of the mean (SEM).

## Results

### Estrogen receptor beta is the predominant form in the human airway epithelium

**Figure 1:**
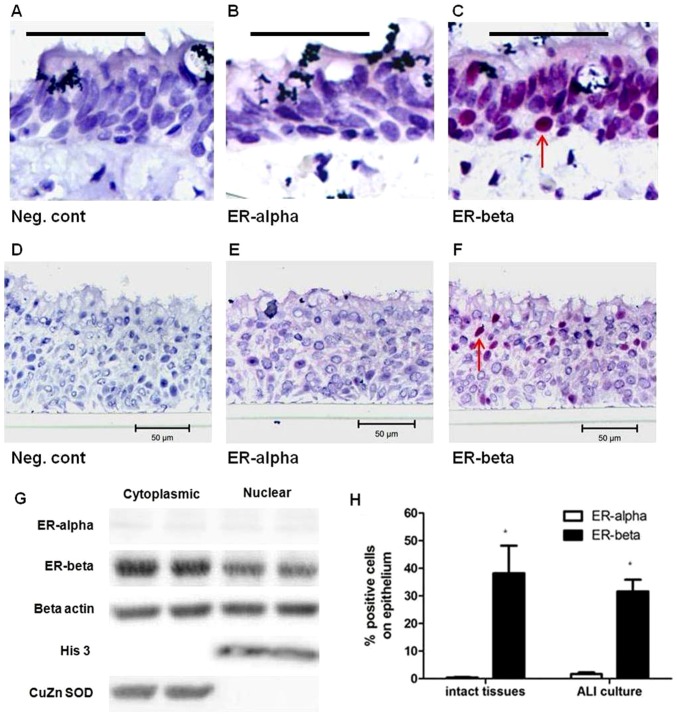
Protein expression of estrogen receptors in excised human large airways of control lungs: A) rabbit immunogobulin-G (IgG) control, B) ER-α and C) ER-β by immunohistochemistry. Protein expression of estrogen receptors in air liquid interface (ALI) cultures: D) rabbit immunogobulin-G (IgG) control, E) ER-α and F) ER-β. G) Western blot (WB) analysis of ER-α and ER-β protein expression in cytoplasmic and nuclear fraction of control ALI cultures at passage 4. Beta-actin, superoxide dismutase (SOD) and histone 3 were used as house-keeping protein, cytoplasmic and nuclear-specific marker, respectively. Representative WB images of N = 4 in duplicate lanes is shown. H) Quantification of positive cell staining of nuclei in red is expressed as a percentage of total cells on the epithelium of intact human airways and in control ALI cultures. Images are representative of N = 4 female excised airways and N = 4 ALI cultures. Sections were counterstained with hematoxylin for nuclei (blue) and positive stains were indicated by red arrows. (5 µm sections; scale bars = 50 µm). *P<0.05 represents statistical significance using a non-parametric t-test.

To confirm the expression of estrogen receptors in the human airway epithelium in human tissues and cells in culture, immunohistochemical staining of ER-α and ER-β was performed on resected human lung tissues with normal lung function and in ALI cultures at passage 4. We first evaluated the expression of the two estrogen receptors ER-α (68 kDa) and ER-β (55 kDa) in the large airways of female subjects in the pre-menopausal age group (15–45 years) with normal lung function (Figure 1A-C) and in ALI cultures (Figure 1D-F) by immunohistochemistry. The demographic characteristics of these individuals are shown in Table S1. Human breast tumour sections were used as positive controls for ER-α and ER-β by immunostaining and breast cancer cell line (Mcf-7) as positive control by western blot (Figure S1 A-D). To confirm the specificity of the ER-α and ER-β antibodies by IHC, we have performed western blot using cytoplasmic and nuclear protein fractions from control ALI cultures. Beta-actin, superoxide dismutase (SOD) and histone 3 were used as house-keeping protein, cytoplasmic and nuclear-specific markers, respectively. Western blot data showed that ER-β protein was the predominant form of estrogen receptor in both cytoplasmic and nuclear compartment of control ALI cultures, with greater expression in the cytoplasm (Figure 1G). This observation was reflected in the quantification of estrogen receptors by immunohistochemistry in figure 1H where ER-β was the predominant form of estrogen receptor with 38.2±9.9% and 31.6±4.3% of epithelial cell population resided in the nuclear compartment by cell counting in intact tissues and ALI cultures, respectively (Figure 1H).

### Estradiol enhances PAS-positive cell count in ALI cultures

**Figure 2:**
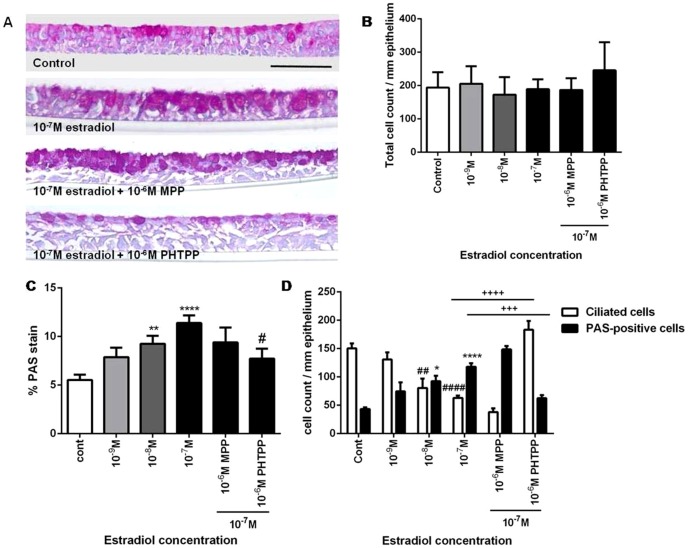
Effect of estradiol on Periodic Acid Schiff (PAS)-positive cell staining in air liquid interface (ALI) cultures. A) ALI cultures (N = 4) were treated with estradiol (vehicle control, 10^−9^, 10^−8^, 10^−7^ M) and ER-α (10^−6^ M MPP) or ER-β (10^−6^ M PHTPP) antagonists for 2 weeks. Images are representative of 4 different donors with 3 biological replicates (5 µm sections; scale bars = 50 µm). B) Total cell count normalized to millimeters of epithelium by manual counting of nuclei counterstained with hematoxylin (N = 4). C) Quantification of the percentage of PAS stain of the entire cross section of the epithelium (**P<0.01, ****P<0.0001 against control, #P<0.05 against 10^−7^ M estradiol-treated group). D) Quantification of cell count for PAS-positive cells and ciliated cells in the apical compartment of the epithelium with increasing estradiol concentration, and in the presence of 10^−6^ M PHTPP or MPP with a single fixed concentration of estradiol at 10^−7^ M (N = 4). * P<0.05, ## P<0.01 and ****/#### P<0.0001 compared against vehicle control, +++ P<0.001 and ++++ P<0.0001 compared against 10^−7^ M estradiol. One-way ANOVA with Bonferroni's test was used in all analyses.

Incubation of mucociliated NHBE cells in ALI with estradiol (in physiologic concentrations) for 2 weeks resulted in a concentration-dependent increase in the total % PAS-stained area, and PAS-positive cell count, which was normalized to the total length of the epithelium in millimeters (Figure 2A; purple color stain indicates PAS-positive cells; scale bar = 50 µm). The total cell count, as measured by the number of nuclei, did not change with all treatment groups (Figure 2B). The % area of PAS staining increased from a baseline control value of 5.5±0.5 to 11.4±0.8 with 10^−7^ M estradiol treatment and reduced to 7.7±1.0 with PHTPP, but not with MPP, at a concentration of 10^−6^ M (Figure 2C). All PAS-positive cells were localized to the apical compartment of the epithelium. Total number of PAS-positive cells on the apical surface was increased from a baseline control value of 43±3 to 92±10 and 118±6 per mm epithelium in the presence of 10^−8^ M and 10^−7^ M estradiol, respectively, and was attenuated with 10^−6^ M PHTPP, but not with 10^−6^M MPP (Figure 2D; black bar  =  PAS-positive cells). ALI cultures treated with 10^−7^ M estradiol resulted in a decrease in total ciliated cell count from 150±9 to 63±4 per mm epithelium, and was restored to baseline control values with 10^−6^ M PHTPP, but not with MPP antagonist (Figure 2D; white bars  =  ciliated cells). Human lung tissue section was used as positive control for PAS-staining (Figure S2 B).

### Estradiol enhances goblet cell count and MUC5AC protein expression in ALI cultures

**Figure 3:**
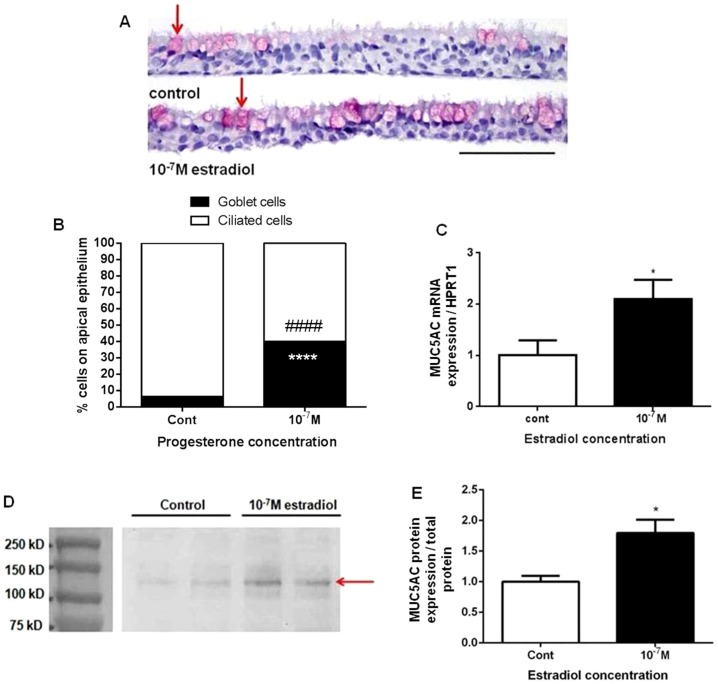
Effects of estradiol on MUC5AC mRNA and protein expression. A) MUC5AC immunostaining in ALI cultures treated with 10^−7^ M estradiol for 2 weeks (goblet cells indicated by red arrows and counterstained with hematoxylin of nuclei). B) Quantification of the number of goblet cells and ciliated cells in the apical compartment of estradiol-treated ALI cultures expressed as a percentage of all cells in the apical compartment. #### P<0.0001 (ciliated cells) and **** P<0.0001 (goblet cells) vs. vehicle control, N = 4. C) MUC5AC mRNA expression normalized to HPRT1 housekeeping using real time PCR. D) MUC5AC protein present in ALI culture secretion was analysed by western blot, normalized by the total volume of apical washes per well and quantified by densitometry of N = 4 donors in duplicate lanes E). All values shown are mean ± SEM of 4 different donors with 3 biological replicates. Non-parametric t-test were used in all analyses.

To further determine whether estradiol-stimulated changes in PAS-positive cells, MUC5AC immunostaining was performed in control and 10^−7^ M estradiol-treated ALI cultures (Figure 3A; red arrow indicates goblet cells; scale bar = 50 µm). Intact human female airway tissue was used as a positive control for MUC5AC protein expression, an indicator for goblet cells (Figure S2 A). In baseline vehicle control cultures, ciliated cells were the predominant cell type and accounted for 93.6±1.1% of epithelial cells, while goblet cells accounted for 6.3±1.1% (Figure 3B). The percentage of goblet cells increased from 6.3±1.1% in vehicle control to 39.9±4.7% in 10^−7^ M estradiol. When cultured in 10^−7^ M estradiol, the percentage of ciliated cells decreased from 93.6±1.1% in vehicle control to 60.1±4.7%. Quantification of mRNA transcripts by quantitative real-time PCR showed that 10^−7^ M estradiol enhanced MUC5AC mRNA expression from 1.0±0.3 to 2.1±0.4 compared to vehicle control (Figure 3C). Estradiol increased MUC5AC protein by 1.8±0.2 fold above vehicle control in apical ALI culture secretion after normalizing by the volume of apical wash per well (Figure 3D, red arrow indicates MUC5AC protein; figure 3E is the densitometry of 3D).

### Estradiol enhanced nuclear factor of activated protein c1 (NFATc1)

**Figure 4:**
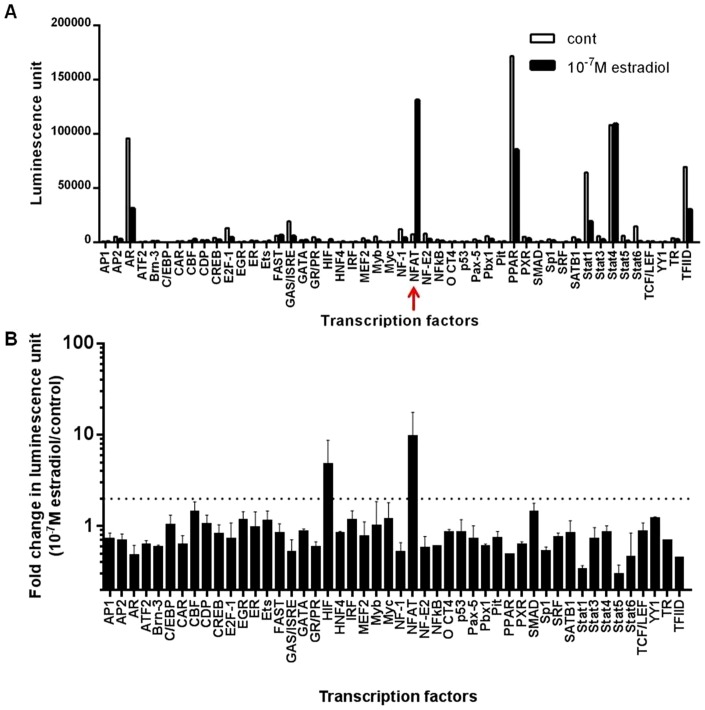
Transcription factor expression panel demonstrates increased total nuclear factor of activated T-cell (NFAT) protein in the nuclear fraction of primary female NHBE cells after 24 h 10^−7^ M estradiol treatment for 24 h. Data are expressed as (A) raw luminescence unit and (B) relative fold increase in luminescence unit over vehicle controls. The dotted line indicates a threshold of a 2-fold increase in protein expression in estradiol-treated cells compared to vehicle controls. NFAT protein is indicated by red arrow. Values shown are mean ± SEM of 2 female NHBE cell donors.

**Figure 5:**
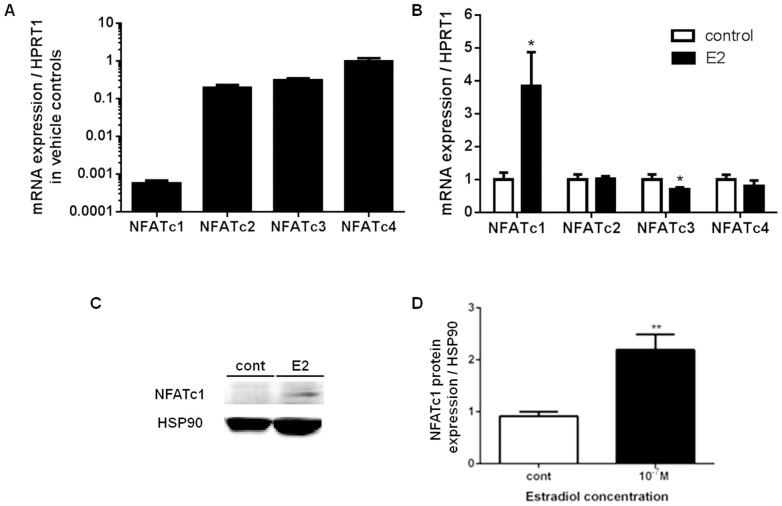
NFAT mRNA and protein expression in ALI cultures of primary NHBEs after 2 weeks of estradiol stimulation at 10^−7^ M. A) Quantitative real-time PCR demonstrates baseline vehicle control mRNA expression of NFAT (c1–c4) expressed as absolute C_t_ value normalized to HPRT1. B) NFATc1–c4 mRNA expression normalized to HPRT1 in vehicle vs. 10^−7^ M estradiol-treated cultures for 2 weeks. C) Western blot showed total NFATc1 protein expression with HSP90 housekeeping protein in vehicle vs. 10^−7^ M estradiol-treated ALI cultures and quantified by densitometry in D) with N = 4. *P<0.05 and **P<0.01 compared against vehicle control using non-parametric t-test and expressed as fold increase over control in B and D.

A transcription factor (TF) activation profiling plate array was used to screen for activated TFs in the nuclear protein extract from primary NHBE cells in monolayer that were treated with 10^−7^ M estradiol for 24 h (Figure 4A-B). The dotted line in figure 4B indicated a threshold of a 2-fold increase in luminescence (estradiol-treated cells over vehicle controls). Estradiol increased the expression of total NFAT protein by 10 fold in the nuclear fraction of NHBE cells after 24 h of incubation. We further investigated the presence of NFAT and its role in regulating MUC5AC mRNA and protein expression with estradiol stimulation in ALI cultures. We showed that ALI cultures expressed all four isoforms of NFAT mRNA: NFATc1, NFATc2, NFATc3 and NFATc4 at baseline levels in vehicle controls with values expressed as absolute change in C_t_ values and normalized to HPRT1 (Figure 5A). Estradiol increased NFATc1 mRNA by 3.8±1.0 fold (Figure 5B), and protein expression by 2.2±0.3 fold over control at 24 h (Figure 5C-D). No change was observed in NFATc2 and NFATc4 mRNA expression with estradiol treatment; however NFATc3 mRNA was attenuated from 1.0±0.2 to 0.7±0.06 fold. Estradiol-stimulated increase in NFATc1 protein in ALI cultures accounted for the total increase in activated NFAT protein in the nuclear extract shown in figure 4.

**Figure 6:**
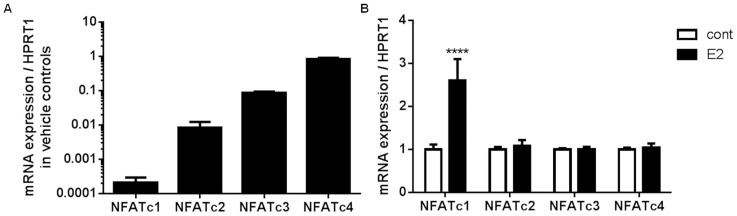
NFAT mRNA and protein expression in 1HAE_0_ cells after estradiol stimulation for 24 h. A) Baseline mRNA expression of NFAT (c1–c4) in 1HAE_0_ cells quantified by real-time PCR expressed as absolute change in C_t_ value normalized to HPRT1. B) NFATc1–c4 mRNA expression normalized to HPRT1 in cells treated with 10^−7^ M estradiol for 24 h. Data are expressed as fold increase over vehicle controls. Values shown are mean ± SEM of experiments performed in 3 independent experiments. ****P<0.0001 compared against vehicle control in each gene using non-parametric t-test in B-C.

**Figure 7:**
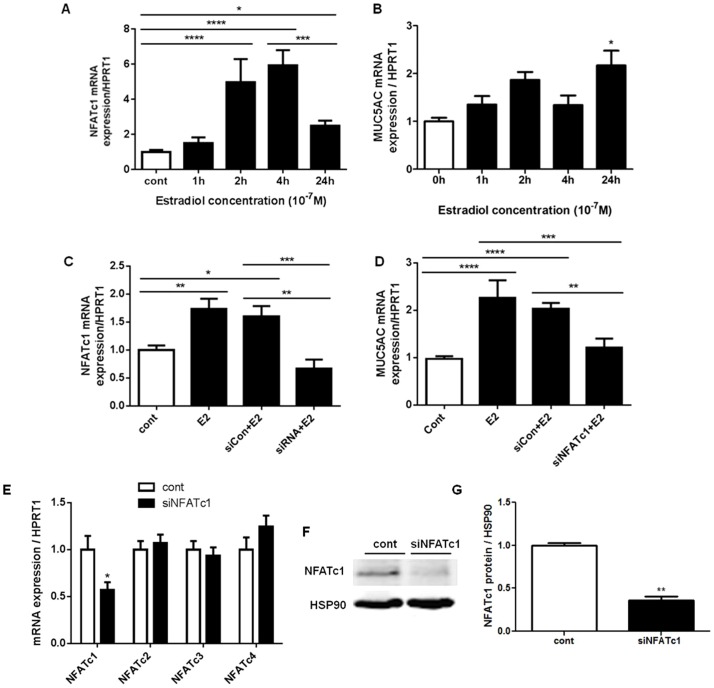
Effect of estradiol and NFATc1-specific siRNA on NFATc1 and MUC5AC mRNA expression in 1HAE_0_ cells. A) Quantitative real-time PCR demonstrates 10^−7^ M estradiol stimulates a time-dependent increase in NFATc1 mRNA/HPRT1 expression with maximal increase at 4 h. B) 10^−7^ M estradiol stimulates a time-dependent increase in MUC5AC mRNA/HPRT1 expression with maximal increase at 24 h. C) 24 h 10^−7^ M estradiol treatment increases NFATc1 mRNA/HPRT1 expression in cells 48 h post-transfection with scrambled control siRNA (siCon+E2) and this effect is reduced by transfection with NFATc1 siRNA (siNFATc1+E2). D) MUC5AC mRNA/HPRT1 expression was increased by 24 h treatment with 10^−7^ M estradiol (E2) with scrambled siRNA transfection (siCon+E2), but was attenuated by transfection with NFATc1 siRNA (siNFATc1+E2). E) NFATc1–c4 mRNA expression in cells treated with 100 nM NFATc1 siRNA for 48 h. F) A representative western blot of NFATc1 protein knockdown expression with 100 nM NFATc1 siRNA treatment for 48 h and quantified by densitometry in G) with N = 3. Values shown are mean ± SEM of experiments performed in 3 independent experiments. *P<0.05, **P<0.01, ***P<0.001 and ****P<0.0001 using one-way ANOVA with Bonferroni's test in A-D and non-parametric t-test was used in E and G.

**Figure 8:**
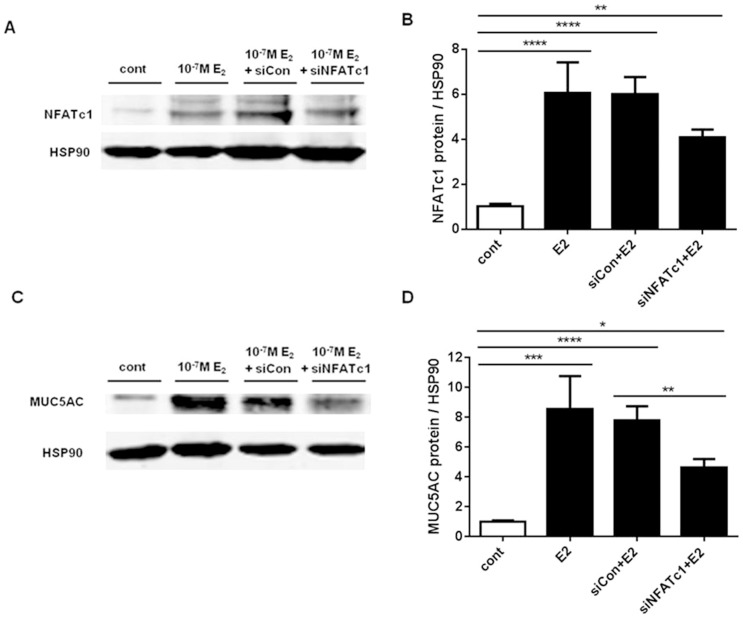
Effect of estradiol and NFATc1-specific siRNA on NFATc1 and MUC5AC protein expression in 1HAE_0_ cells by western blot. A-B) 24 h 10^−7^ M estradiol treatment alone (E2) and 48 h post-transfection with scrambled siRNA (siCon+E2) increases total cellular NFATc1 protein expression (normalised to HSP90). The effect of E2 is attenuated in cells transfected with siRNA against NFATc1 (siNFATc1+E2). C-D) Total cellular MUC5AC protein/HSP90 expression was increased by 10^−7^ M estradiol alone (E2) and in scrambled siRNA controls (siCon+E2), but was attenuated by transfection with NFATc1 siRNA (siNFATc1+E2) treatment. Values shown are mean ± SEM of experiments performed in 3 independent experiments. *P<0.05, **P<0.01, ***P<0.001 and ****P<0.0001 using one-way ANOVA with Bonferroni's test in all analyses.

In order to demonstrate whether NFATc1 plays any role in regulating MUC5AC mRNA expression, we used 1HAE_0_ cells as a model. Baseline mRNA expression levels of NFATc1–c4 in control 1HAE_0_ cells are shown in Figure 6A. 10^−7^ M estradiol treatment enhanced NFATc1, but not NFATc2–c4, mRNA expression by ∼2.5 fold after 24 h (Figure 6B). We further showed a time-dependent increase in NFATc1 mRNA with 10^−7^ M estradiol, which peaked at 4 h with a 5.9±0.9 fold increase and declined to 2.5±0.3 fold above control at 24 h (Figure 7A). A time-dependent increase in MUC5AC mRNA was also observed with estradiol stimulation, which peaked at 24 h with a 2.2±0.3 fold increase above control (Figure 7B). We silenced NFATc1 with siNFATc1 for 48 h prior to the addition of 10^−7^ M estradiol for 24 h and observed changes in MUC5AC mRNA expression. 10^−7^ M estradiol (E2) and siCon+E2 increased NFATc1 mRNA by 1.7±0.2 and 1.6±0.2 fold over control, respectively (Figure 7C). The use of siNFATc1 in the presence of estradiol attenuated NFATc1 mRNA to 0.7±0.2 fold below the control group with statistical comparison with the siCon+E2 treated group. 10^−7^ M estradiol (E2) and siCon+E2 increased MUC5AC mRNA by 2.3±0.4 and 2.0±0.1 fold over control, respectively (Figure 7D). MUC5AC mRNA levels were attenuated by siNFATc1 in the presence of estradiol to 1.2±0.2 fold above the control group with statistical comparison with siCon+E2 treated group. NFATc1 siRNA alone reduced NFATc1 mRNA expression by ∼50%, while NFATc2–c4 mRNA expression was unchanged after 48 h of siNFATc1 treatment (Figure 7E). 100 nM NFATc1 siRNA treatment in 1HAE_0_ cells for 48 h significantly reduced NFATc1 protein expression by ∼70% (control: 1.0±0.03 and siNFATc1: 0.3±0.05) after normalization to HSP90 (Figure 7F-G). Furthermore, we showed that 10^−7^ M estradiol (E2) and siCon+E2 increased total cellular NFATc1 protein by 6.0±0.8 and 6.0±0.8 fold over control (Figure 8A-B). The use of siNFATc1 attenuated estradiol-stimulated NFATc1 protein to 4.1±0.4 fold over control. Similarly, 10^−7^ M estradiol (E2) and siCon+E2 increased total MUC5AC protein levels by 8.5±2.2 and 7.8±0.9 fold over control. This was attenuated to 4.6±0.6 fold over control with siNFATc1 (Figure 8C-D). Collectively, these data suggest that NFATc1 is a novel regulator of estradiol-mediated MUC5AC mRNA expression.

### Estradiol enhances fucosyltransferase mRNA expression and total protein fucosylation

**Figure 9:**
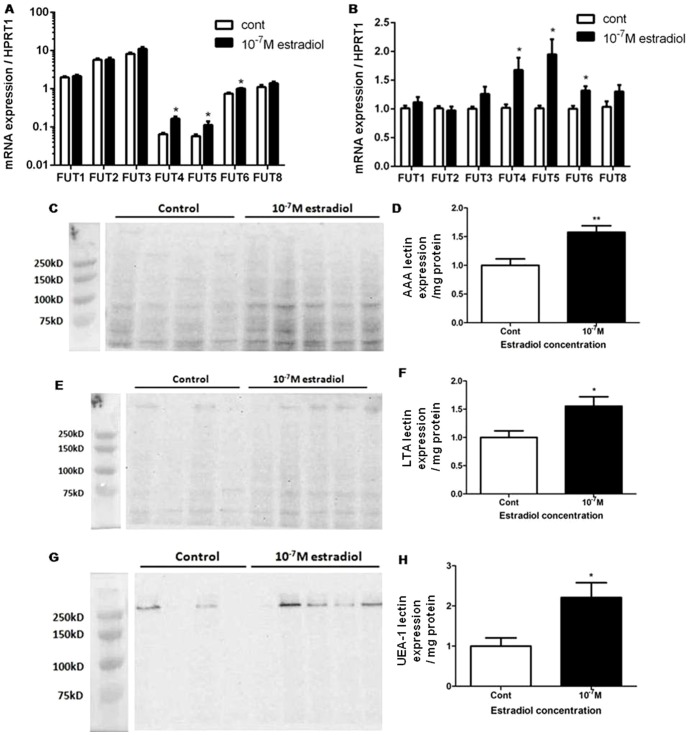
Effects of estradiol on fucosyltransferase mRNA expression in ALI cultures. 10^−7^ M estradiol (black bars) increased FUT-4, -5 and -6 mRNA expression compared with vehicle control (white bars). Data are expressed as A) absolute change in C_t_ value normalized to HPRT1 and B) fold increase in mRNA expression over vehicle control normalized using HPRT1. Values shown are mean ± SEM of 4 different donors performed in 3 biological replicates. Total fucose sugar residues in the cytoplasmic protein fractions of ALI cultures were determined by lectin binding assays. Fucose residues were significantly increased with 10^−7^ M estradiol using fucose binding lectins, C-D) AAA, E-F) LTA and G-H) UEA-1. D, F, and H are densitometric quantifications of C, E, and G, respectively. The intensity of all bands in each lane in detecting total fucose residues were quantified using the software program Image J and normalized to total protein loaded per lane in milligrams. Data is expressed as fold increase over vehicle control. Values shown are mean ± SEM of experiments performed with N = 4 donors. * P<0.05, ** P<0.01 compared against vehicle control. Non-parametric t-tests were used in all statistical analyses.

The synthesis of mature mucin requires post-translational modification by unique glycosyltransferases in the endoplasmic reticulum [19]. To elucidate the specific enzymes responsible for the observed increase in estradiol-stimulated PAS staining, we have measured fucosyltransferase mRNA (FUT-1 to -9) [19] that were known to be expressed in the lungs. All of the above, except FUT-7/9, were detectable in ALI cultures by real time PCR and expressed as both absolute change in C_t_ values normalized to HPRT1 and relative fold increase with estradiol treatment compared to vehicle controls (Figure 9A-B). 10^−7^ M concentration of estradiol increased FUT-4, -5, and -6 mRNA expressions by 1.7±0.2, 2.0±0.3 and 1.3±0.08 fold over vehicle control. To determine whether estradiol-stimulated changes in glycosyltransferase mRNA expression were associated with alterations in specific sugar glycosylation pattern, lectin assays (AAA, LTA and UEA-1 for detecting α-1,2 fucose residues) were used. Treatment of ALI cultures with 10^−7^ M estradiol increased total α-1,2 fucose residues by 1.6±0.1, 1.5±0.2 and 2.1±0.3 fold over vehicle control with AAA (Figure 9C-D), LTA (Figure 9E-F) and UEA-1 (Figure 9G-H) lectins, respectively. An overall increase in fucosylation by FUT-4, -5 and -6 in the cytoplasmic fraction partially explained the observed increase in PAS-positive cell staining.

### Cell proliferation and cytotoxicity

To evaluate whether estradiol alters cell proliferation, Ki67 immunostaining in ALI culture was assessed (Figure S3 A). 10^−7^ M estradiol increased the percentage of Ki67-positive nuclei in the basal lateral compartment of control sections from a baseline value of 2.0±0.4% to 4.2±0.6% (Figure S3 B). To determine whether estrogen receptor antagonists induced cellular cytotoxicity, TACS DAB in-situ apoptosis detection system was used to detect DNA breaks in apoptotic cells of paraffin-embedded ALI culture sections. TACS nuclease was used on ALI sections as positive control in generating an artificial DNA break (data not shown). Treatment of ALI culture with 10^−7^ M estradiol for 2 weeks in the presence of ER antagonists (10^−6^ M MPP or 10^−6^ M PHTPP) revealed no significant increase in the % of apoptotic cells (Figure S3 C). Supernatant from 10% cigarette smoke extract (CSE)-treated 1HAE_0_ cells for 24 h was used as a reference positive control for the LDH assay (Figure S4 A-B). Trypan blue stained 1HAE_0_ cell nuclei black, which confirmed cellular cytotoxicity. LDH assay revealed no significant change in % cellular cytotoxicity in 1HAE_0_ cells treated with 100 mM siNFATc1 and/or 10^−7^ M estradiol (Figure S4 C).

## Discussion

Mucus hypersecretion with plugging of airways is one pathological link that is common to most chronic inflammatory airway disease (e.g. asthma, chronic obstructive pulmonary disease-COPD, CF) [3]. As the predominant form of mucin in the human airway, MUC5AC is produced mainly in goblet cells in the surface epithelium [6]. In this study, we used a fully differentiated cell culture model with pseudostratified phenotype in an air liquid interface and showed that estradiol increases both mRNA and protein expression of MUC5AC and modifies glycosylation of mucins in human airways. Additionally, we found that this effect is largely mediated by estrogen receptor beta. Inhibition of estrogen receptor beta by 10^−6^ M PHTPP attenuated estradiol-related increase in goblet cells in the bronchial epithelium.

To further characterize the mechanism by which estradiol regulates mucin, we performed protein microarray analysis consisting of a panel of transcription factors. Total activated nuclear factor of activated T-cell (NFAT) was increased in the nuclear fraction of NHBE cells after estradiol stimulation for 24 h. Although NFAT protein was originally discovered in T-cells, studies have shown that they are expressed in the human airway epithelium and possess some interesting functional properties. For instance, Dave et al. showed that NFATc3 is a direct activator of surfactant protein A, B, and C, and Forkhead box protein A1 and A2 genes in the airway epithelium during lung maturation [20]. NFATc3 has also been shown to directly interact with thyroid transcription factor-1 in lung epithelial cells to regulate surfactant protein D gene [21]. In our study, NFATc1, NFATc2, NFATc3 and NFATc4 mRNA were confirmed to be expressed in both ALI cultures and in 1HAE_0_ cells. We showed that NFATc1 and MUC5AC mRNA and protein expression were increased with estradiol in both ALI cultures and 1HAE_0_ cells. To determine whether NFATc1 plays a role in regulating MUC5AC mRNA expression, we silenced NFATc1 in 1HAE_o_ cells with siNFATc1. siNFATc1 blunted estradiol-related increases in NFATc1 and MUC5AC mRNA and protein expression. Collectively, these data demonstrate that NFATc1 regulates estradiol-mediated mucus production in human bronchial epithelium. Additional studies will be needed to pinpoint the precise mechanisms by which NFATc1 modifies MUC5AC in the presence of estradiol.

In addition to the regulation of MUC5AC mRNA and protein synthesis, our findings indicate that estrogens affect post-translational modification of mucin. In our ALI model, we observed an increase in the mRNA expression of fucosyltransferase as well as total fucose residues in the cytoplasmic fraction of ALI using three different lectins (UEA-1, AAA and LTA) that specifically bind to the same α-1,2 fucose residues present on all protein. How estrogens modify fucosylation is uncertain. Estradiol may directly increase the expression of fucosyltransferase or indirectly by increasing the number of goblet cells containing these enzymes. Additional studies will be needed to unravel the underlying mechanisms for this observation.

The clinical relevance of increased protein glycosylation of mucin is uncertain. It is now well known that post-translational modification of glycoprotein with fucose, for instance, is essential for normal embryonic growth and development, fertility, and immune function [22]. Alterations in the expression of fucosylated glycans and their cognate fucosyltransferases have been observed in multiple pathologic processes involving inflammation, cancer, and numerous oncogenic events involving signaling events by the Notch receptor family [23]. Moreover, opportunistic bacteria often use lectins (which are protein receptors with high specificity for glycoconjugates) to recognize and adhere to human tissues [15]. Fucosylated glycoconjugates are present in high quantity in CF lungs and may be a target of lectins from pathogenic bacteria such as *Pseudomonas aeruginosa* [15]. This may increase the risk of infection by *Pseudomonas aeruginosa* in the context of CF.

It is important to note several limitations in our current study. First, the ALI culture in our model was not exposed to the fluctuating concentrations of estradiol over a period of 28 days that resembled the female menstrual cycle. In our current model, we used fixed concentrations of estradiol to examine the regulation of MUC5AC mRNA and protein expression. Second, for clinical relevance, we only used female NHBE cells to model the increased level of estrogen in pre-menopausal period. Male NHBE cells in ALI also express similar levels of estrogen receptors with ER-β predominance (data not shown), but we do not know if they have similar regulatory mechanisms related to estradiol as female ALI cultures. Third, although the hormone receptor antagonists we employed have high levels of specificity [24], [25], there may be minor off-target effects that could not be fully accounted for in our analysis. Fourth, it is possible that estradiol may fundamentally alter cellular proliferation of the entire epithelial layer. To determine whether estradiol altered cell proliferation, we stained the ALI cultures with Ki67. It was reassuring that estradiol treatment did not change the total cell numbers and did not induce cellular apoptosis. It only slightly increased Ki67 cell positivity, suggesting a slight prolongation of cell cycle. Finally, while we measured total mucus expression, we did not evaluate other aspect of mucus homeostasis in airways including viscoelastic properties of mucus, mucus clearance, and the effect of mucus on airway resistance.

Notwithstanding these limitations, our data are relevant to chronic airway diseases such as asthma and cystic fibrosis pathogenesis because an increase in goblet cells is associated with mucus plugging in the small airways, which has been shown to be the single most important pathologic predictor of increased mortality in severe COPD [3]. Our study demonstrates that estrodiol may play an important role in modulating mucus expression in the human airway epithelium, which may impact on disease expression in women who are susceptible to chronic airway diseases.

## Supporting Information

Figure S1 A) rabbit immunogobulin-G (IgG) control, B) ER-α and C) ER-β immunostaining in human breast tissue section as positive control and counterstained by hematoxylin. Scale bars = 50 µm. D) ER-α and ER-β protein expression in breast cancer cells (MCF-7) as positive control by WB.
(TIF)

Figure S2 A) MUC5AC immunostaining in human lung tissues as positive control for MUC5AC staining (indicated by red color; scale bar = 50 µm) with hematoxylin as counterstain. B) PAS-staining in human lung tissues as positive control for PAS-staining (indicated by purple color; scale bar = 50 µm) and counterstained by hematoxylin.
(TIF)

Figure S3 A) Cell proliferation in ALI cultures was assessed by Ki-67 staining. B) Estradiol enhanced Ki67-positive cell staining in the basal epithelium. C) Quantification of % apoptotic cells by cell counting in ALI cultures counter-stained with methyl green for nuclei. Images are representative of 4 different donors. **P<0.01 represents statistical significance compared against control using non-parametric t-test in B. One-way ANOVA with Bonferroni's multiple comparisons test was used in C.
(TIF)

Figure S4 Lactate dehydrogenase (LDH) assay was used to measure cellular cytotoxicity. 1HAE_0_ cells were exposed to a final concentration of 10% CSE for 24 h and cell supernatant was used as positive control for LDH assay. A-B) A final concentration of 0.2% Trypan blue solution was added to cells as an indication of cell death. (Scale bar = 50 µm). C) % cell cytotoxicity ( = 100*[A_exp. value_- A_low control_]/[A_high control_-A_low control_], where A = absorbance at 492 nm) in cells treated with siNFATc1 and/or estradiol were normalized to LDH level secreted by cells that were exposed to 10% CSE (high control). One-way ANOVA with Bonferroni's multiple comparisons test was used in C.
(TIF)

Table S1 Demographics of subjects from figure 1A-C for estrogen receptor expression by immunohistochemistry.
(TIF)
