# Supplementary figures and images for: Estradiol Increases Mucus Synthesis in Bronchial Epithelial Cells
Source: PLoS One. 2014 Jun 25;9(6):e100633. doi: 10.1371/journal.pone.0100633 (PMC4070981; doi:10.1371/journal.pone.0100633)

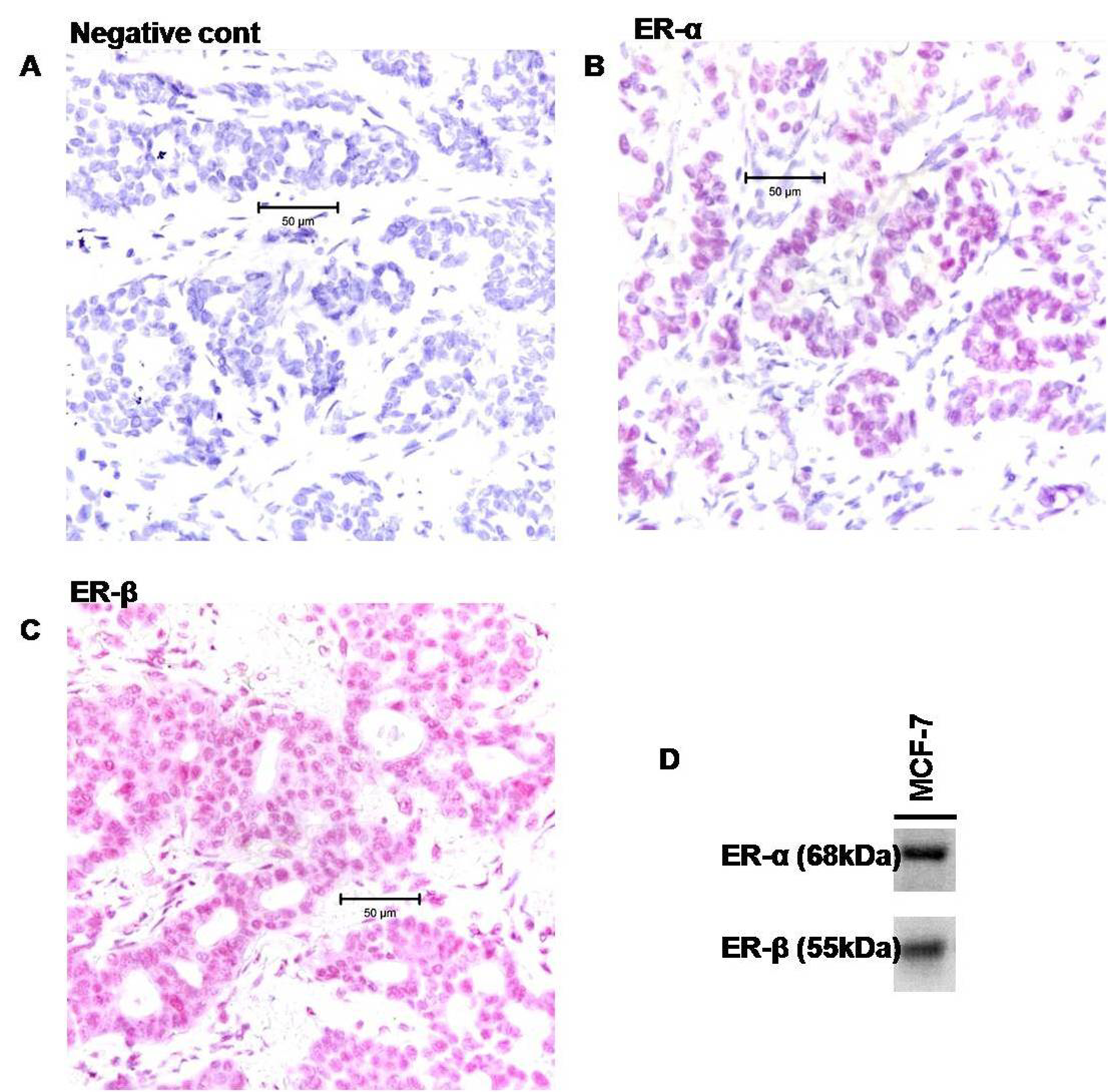

Supplement: Figure S1 — A) rabbit immunogobulin-G (IgG) control, B) ER-α and C) ER-β immunostaining in human breast tissue section as positive control and counterstained by hematoxylin. Scale bars = 50 µm. D) ER-α and ER-β protein expression in breast cancer cells (MCF-7) as positive control by WB. (TIF) [file pone.0100633.s001.tif]

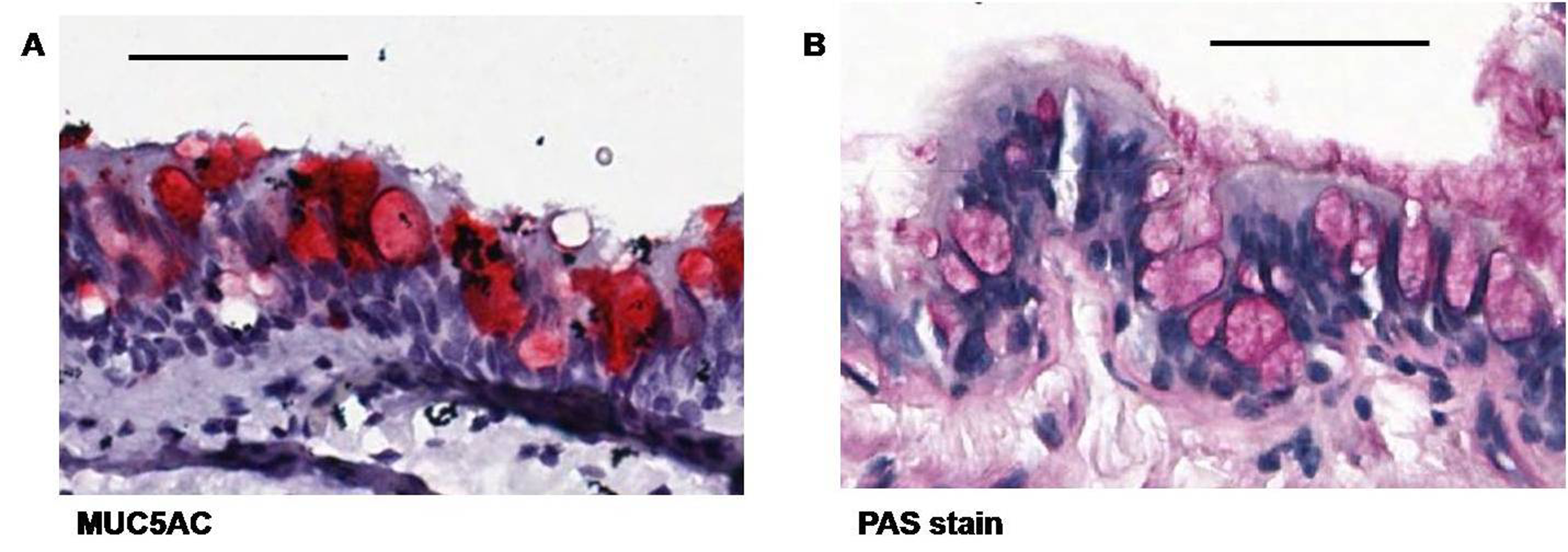

Supplement: Figure S2 — A) MUC5AC immunostaining in human lung tissues as positive control for MUC5AC staining (indicated by red color; scale bar = 50 µm) with hematoxylin as counterstain. B) PAS-staining in human lung tissues as positive control for PAS-staining (indicated by purple color; scale bar = 50 µm) and counterstained by hematoxylin. (TIF) [file pone.0100633.s002.tif]

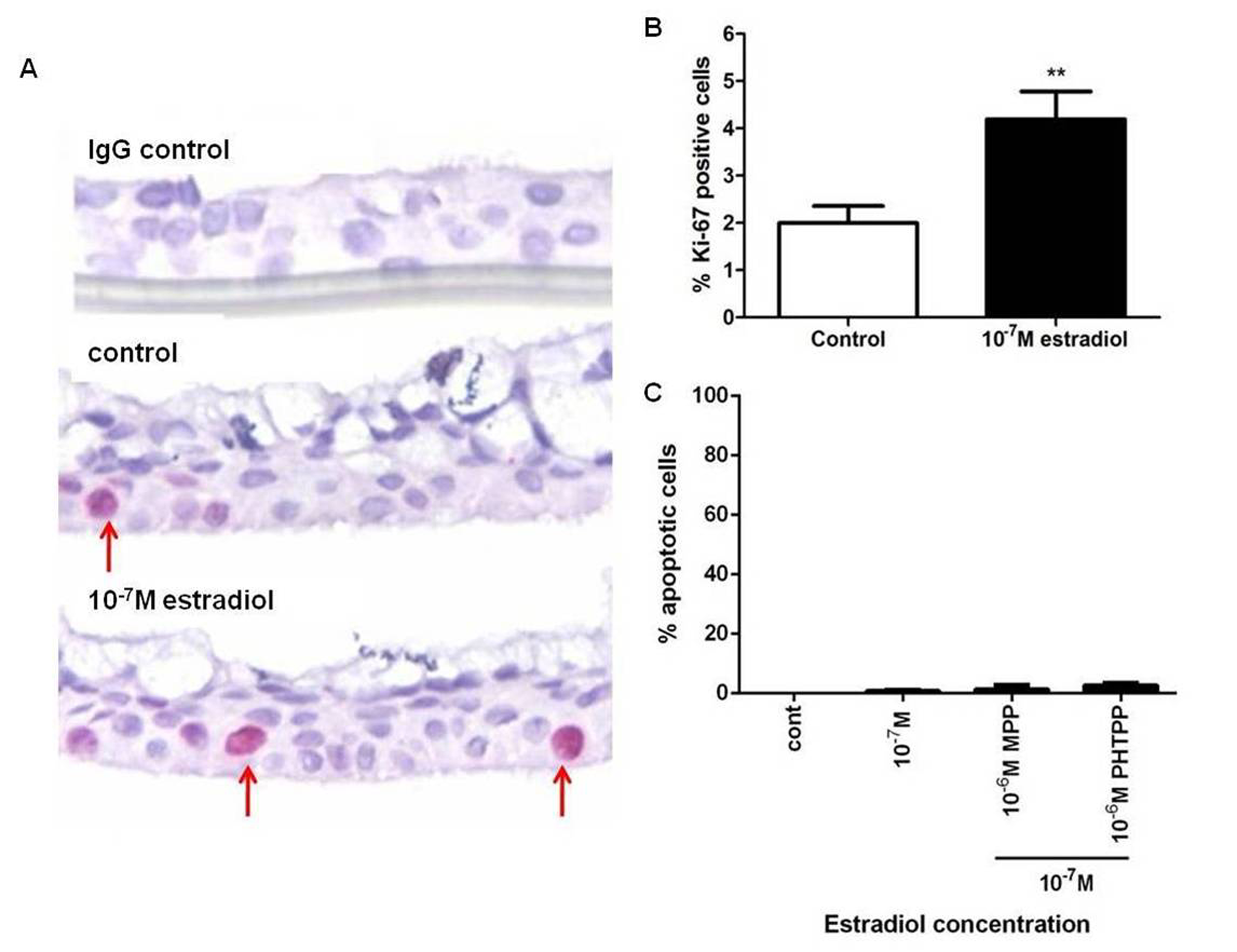

Supplement: Figure S3 — A) Cell proliferation in ALI cultures was assessed by Ki-67 staining. B) Estradiol enhanced Ki67-positive cell staining in the basal epithelium. C) Quantification of % apoptotic cells by cell counting in ALI cultures counter-stained with methyl green for nuclei. Images are representative of 4 different donors. **P<0.01 represents statistical significance compared against control using non-parametric t-test in B. One-way ANOVA with Bonferroni's multiple comparisons test was used in C. (TIF) [file pone.0100633.s003.tif]

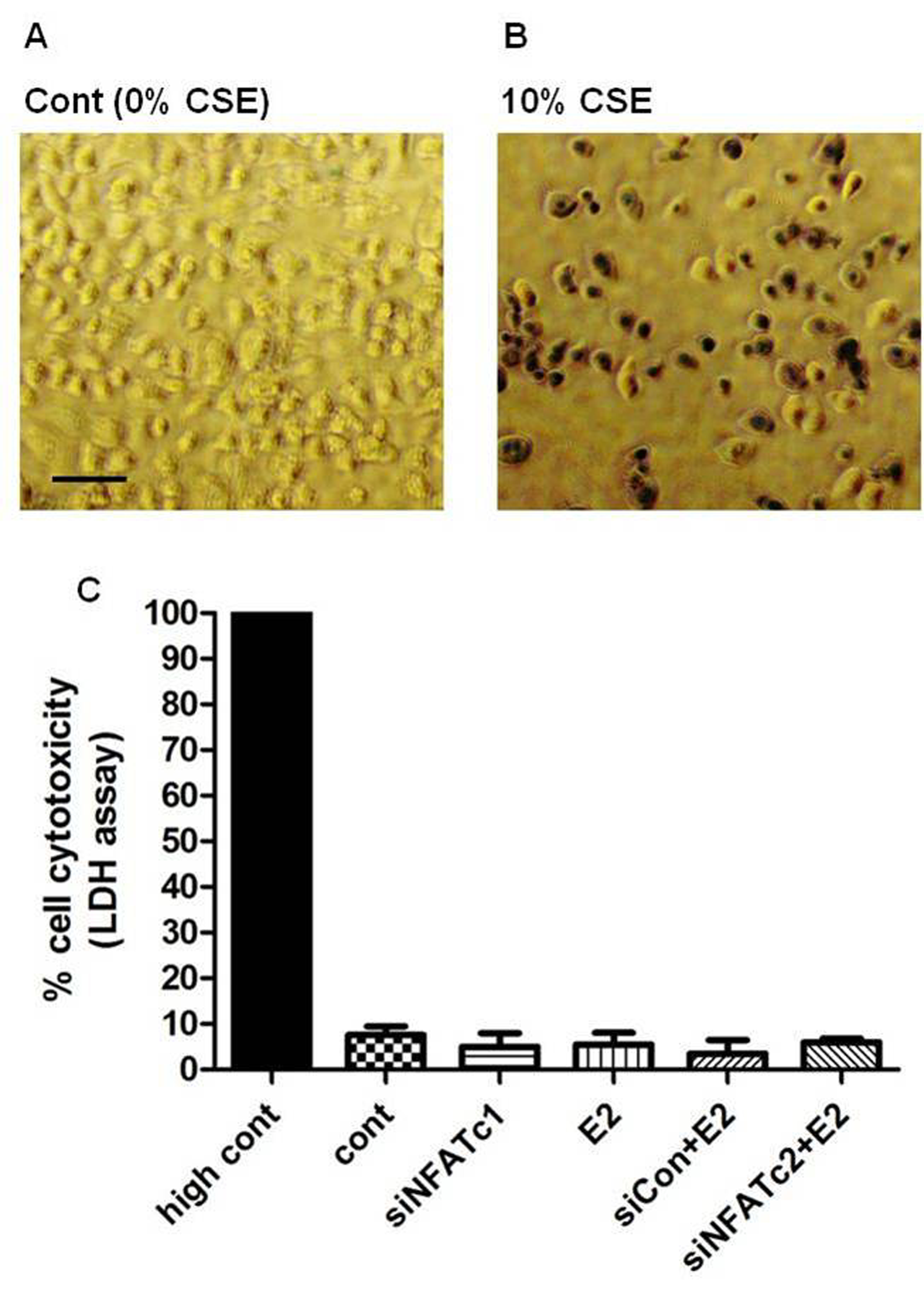

Supplement: Figure S4 — Lactate dehydrogenase (LDH) assay was used to measure cellular cytotoxicity. 1HAE0 cells were exposed to a final concentration of 10% CSE for 24 h and cell supernatant was used as positive control for LDH assay. A-B) A final concentration of 0.2% Trypan blue solution was added to cells as an indication of cell death. (Scale bar = 50 µm). C) % cell cytotoxicity ( = 100*[Aexp. value- Alow control]/[Ahigh control-Alow control], where A = absorbance at 492 nm) in cells treated with siNFATc1 and/or estradiol were normalized to LDH level secreted by cells that were exposed to 10% CSE (high control). One-way ANOVA with Bonferroni's multiple comparisons test was used in C. (TIF) [file pone.0100633.s004.tif]

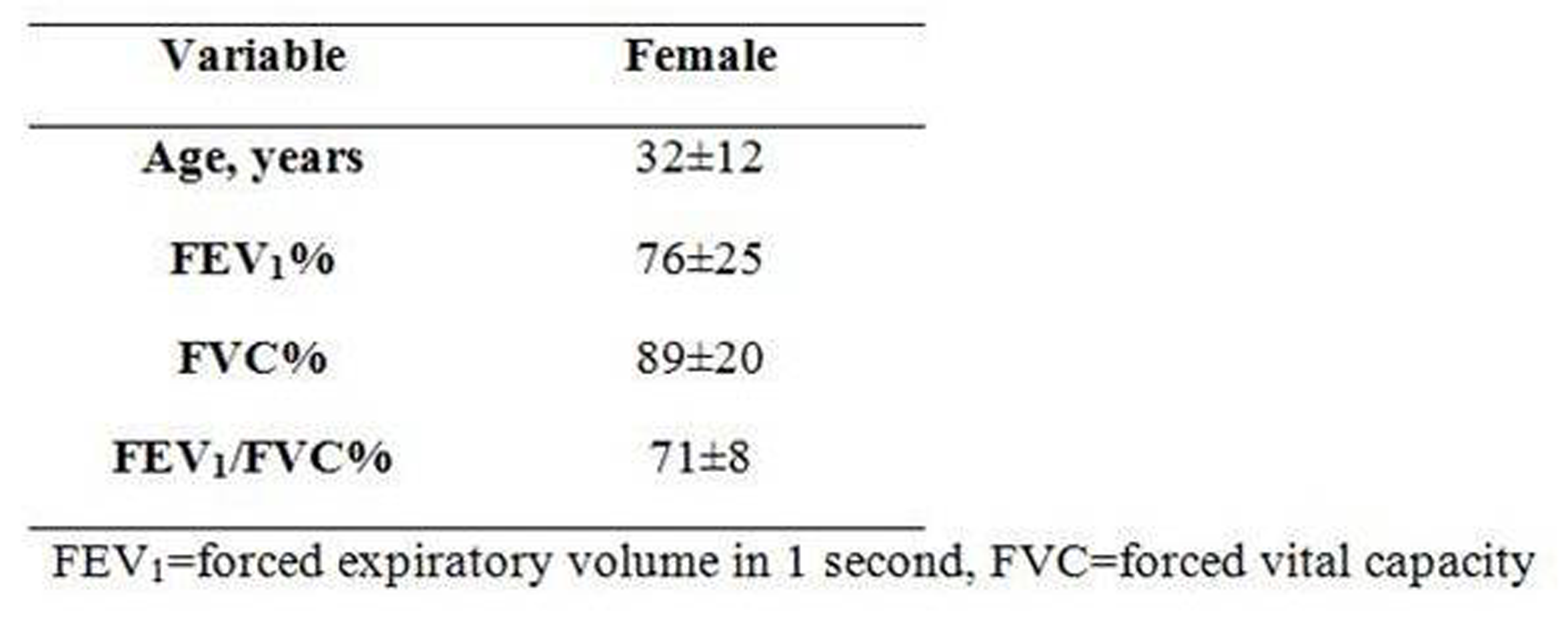

Supplement: Table S1 — Demographics of subjects from figure 1A-C for estrogen receptor expression by immunohistochemistry. (TIF) [file pone.0100633.s005.tif]
